# Brain-cognition relationships in late-life depression: a systematic review of structural magnetic resonance imaging studies

**DOI:** 10.1038/s41398-023-02584-2

**Published:** 2023-08-19

**Authors:** Tulip Marawi, Nicholas J. Ainsworth, Peter Zhukovsky, Neda Rashidi-Ranjbar, Tarek K. Rajji, Maria Carmela Tartaglia, Aristotle N. Voineskos, Benoit H. Mulsant

**Affiliations:** 1 Institute of Medical Science, Temerty Faculty of Medicine, University of Toronto, Toronto, ON Canada; 2 Campbell Family Mental Health Research Institute, Centre for Addiction and Mental Health, Toronto, ON Canada; 3 Department of Psychiatry, Temerty Faculty of Medicine, University of Toronto, Toronto, ON Canada; 4 Keenan Research Centre for Biomedical Science, Li Ka Shing Knowledge Institute, Toronto, ON Canada; 5 Toronto Dementia Research Alliance, University of Toronto, Toronto, ON Canada; 6 Department of Neurology, Temerty Faculty of Medicine, University of Toronto, Toronto, ON Canada; 7 Tanz Centre for Research in Neurodegenerative Diseases, University of Toronto, Toronto, ON Canada

**Keywords:** Depression, Learning and memory

## Abstract

**Background:**

Most patients with late-life depression (LLD) have cognitive impairment, and at least one-third meet diagnostic criteria for mild cognitive impairment (MCI), a prodrome to Alzheimer’s dementia (AD) and other neurodegenerative diseases. However, the mechanisms linking LLD and MCI, and brain alterations underlying impaired cognition in LLD and LLD + MCI remain poorly understood.

**Methods:**

To address this knowledge gap, we conducted a systematic review of studies of brain-cognition relationships in LLD or LLD + MCI to identify circuits underlying impaired cognition in LLD or LLD + MCI. We searched MEDLINE, PsycINFO, EMBASE, and Web of Science databases from inception through February 13, 2023. We included studies that assessed cognition in patients with LLD or LLD + MCI and acquired: (1) T1-weighted imaging (T1) measuring gray matter volumes or thickness; or (2) diffusion-weighted imaging (DWI) assessing white matter integrity. Due to the heterogeneity in studies, we only conducted a descriptive synthesis.

**Results:**

Our search identified 51 articles, resulting in 33 T1 studies, 17 DWI studies, and 1 study analyzing both T1 and DWI. Despite limitations, reviewed studies suggest that lower thickness or volume in the frontal and temporal regions and widespread lower white matter integrity are associated with impaired cognition in LLD. Lower white matter integrity in the posterior cingulate region (precuneus and corpus callosum sub-regions) was more associated with impairment executive function and processing speed than with memory.

**Conclusion:**

Future studies should analyze larger samples of participants with various degrees of cognitive impairment and go beyond univariate statistical models to assess reliable brain-cognition relationships in LLD.

## Introduction

Late-life depression (LLD) is typically defined as major depressive disorder (MDD) occurring in adults 60 years or older. Estimates of the prevalence of LLD range from 2–5% among community-dwelling older adults, reaching up to 12% among hospitalized older adults [1]. Most patients with LLD present with more cognitive impairment than age-matched non-depressed controls, and about one-third meet diagnostic criteria for mild cognitive impairment (MCI) [2]. While cognitive deficits associated with LLD can involve any domain of cognition [3, 4], executive function and processing speed are considered to be the core cognitive deficits in LLD [5, 6] and may mediate deficits in other cognitive domains [3, 7, 8]. LLD is also associated with an increased risk of developing dementia of all causes and Alzheimer’s dementia (AD) in particular [9]. Patients with both LLD and MCI (LLD + MCI) have two risk factors for developing AD and therefore could be at an increased risk. LLD can occur first later in life after the age of 60 and is then referred to as late-onset depression (LOD), or it can have occurred first early in life and recurred in late life and is then referred to as early-onset depression (EOD). Both LOD and EOD have been associated with an elevated risk for AD [10]. Several meta-analyses have confirmed that EOD is a risk factor for all-cause dementia and AD [9, 11, 12]. Compared to EOD, LOD has been associated with more severe cognitive impairment [13, 14], and with white matter hyperintensities and other cerebrovascular abnormalities that are common in AD [15]. This led some studies to suggest that LOD is an early neuropsychiatric manifestation of AD [10].

Some of the neurobiological mechanisms explaining the relationship between LLD, MCI, and AD have been summarized through two pathways. First, models of the vascular pathway suggest that cerebrovascular disease, and ischemic lesions in particular, lead to executive dysfunction. Second, models of the inflammation pathway suggest that elevated levels of stress hormones promote neurodegeneration, particularly hippocampal volume loss, thus leading to impaired episodic memory [16]. Other mechanisms linking LLD and MCI include Alzheimer’s pathology (e.g., amyloid beta accumulation) in brain regions related to mood regulation serving as a contributor to depression [17, 18], and cerebral blood flow reductions in brain regions related to mood and cognitive symptoms [19]. A comprehensive discussion of the mechanisms linking LLD and AD is beyond the scope of this review and is the topic of other reviews [16, 17, 19, 20].

Several neuroimaging studies have identified brain structural alterations associated with LLD or LLD + MCI that contribute to the risk of AD [21–23]. However, the literature exploring the association between structural abnormalities and cognitive impairment in LLD and LLD + MCI remains sparse. To address this gap, we conducted a systematic review of studies of brain-cognition relationships in LLD or LLD + MCI. We aimed to identify vulnerable circuits underlying impaired cognition in LLD or LLD + MCI and reveal risk mechanisms for AD. We focused on T1-weighted imaging studies of gray matter (GM) assessing brain volume and thickness and on diffusion-weighted imaging (DWI) studies assessing white matter (WM) measures such as fractional anisotropy (FA) and mean diffusivity (MD). In secondary analyses, we explored whether brain-cognition relationships found across all studies are also present in LLD + MCI, as well as early and late-onset depression subgroups.

## Methods

### Registration

This systematic review was conducted in accordance with the updated Preferred Reporting Items for Systematic Reviews and Meta-Analyses (PRISMA 2020) [24] and registered on the International Prospective Register of Systematic Reviews (PROSPERO: CRD42022292905). The search strategy and protocol were reviewed by a librarian at the Center for Addiction and Mental Health (CAMH), Toronto, Canada, prior to registration and screening.

### Information sources and search strategy

A systematic review of the literature was conducted using MEDLINE, PsycINFO, EMBASE, and Web of Science electronic databases from inception through February 13, 2023. Comprehensive search strategies adapted for Medline, EMBASE, PsycINFO, and Web of Science are available in Supplementary Materials. In brief, our search strategy included Medical Subject Headings (MeSH) and keywords related to three broad search blocks: geriatric depression (age group and condition being studied), structural magnetic resonance imaging (MRI, methodology of interest), and cognition (primary outcome measure). In addition to searching for articles of interest, reference lists of relevant review articles were also searched for additional eligible studies.

### Eligibility criteria and study selection

Studies were included if they met the following criteria: (1) published in a peer-reviewed journal in the English language; (2) participants were older adults aged 55 years or older OR had a mean age of at least 65 years; (3) in at least one group, participants were formally diagnosed with MDD according to criteria from the Diagnostic and Statistical Manual of Mental Disorders (DSM) or International Classification of Diseases (ICD) (regardless of whether they also have MCI); (4) study assessed cognitive performance in diagnostic groups using at least one cognitive measure; (5) study acquired one (or both) of the following structural MRI modalities: T1-weighted scans measuring GM structure (i.e., volume or thickness) or DWI scans measuring WM integrity; and (6) study reported the results of some analysis of the relationship between the imaging measures and cognitive performance.

Studies were excluded if they: (1) focused exclusively on dementia or MCI; (2) exclusively studied depressive symptoms (i.e., without a formal diagnosis of MDD); (3) in the MDD group, included participants diagnosed with a major neurological illness (e.g., stroke, Parkinson’s disease, epilepsy, multiple sclerosis, traumatic brain injury); (4) in the MDD group, included participants with psychotic depression (i.e., did not separate those with MDD with or without psychotic features); (5) reported on case studies or non-human subjects. Conference abstracts, commentaries, opinion pieces, letters to the editor, and reviews were also excluded. As we conducted the review, we added an additional exclusion criterion to exclude clinical trials including participants who received electroconvulsive therapy (ECT) less than 3 months from the time of cognitive testing, due to potential effects of recent ECT on cognitive performance [25]. If ECT was administered more than 3 months prior to the testing date, the study was included. However, no study met such criteria and therefore all ECT studies were excluded.

### Data selection

In accordance with the PRISMA guidelines, studies identified through searching the electronic databases first underwent title and abstract screening by one independent reviewer (TM) to determine their relevance with respect to the population, condition, methodology, and outcomes of interest. After removing any duplicates, full-text review of studies included from the screening stage was conducted. Two independent reviewers (TM, NJA) conducted the full-text review, and disagreements were resolved by a third study team member (BHM). Screening and study selection were conducted using the Covidence reference management system.

### Data extraction

Data from studies meeting the eligibility criteria were extracted and entered into a database including bibliographic information, study type, sample size, mean age of the groups, age of depression onset, depression status, diagnostic criteria, reported treatment, categorical results of all cognitive assessments (e.g., domains with impaired cognition), imaging modality, imaging analysis and processing approach, statistical analysis methods, regions/tracts assessed, and a summary of study findings. Information was recorded for the LLD group and other additional groups (i.e., MCI, LLD + MCI, healthy controls - HC).

### Risk of bias assessment

Quality assessment of all studies included was completed using a modified version of the Newcastle-Ottawa Scale (NOS) reported elsewhere [22]. In summary, points were allocated to each study and summed up to range from 0–8, with scores between 0-3 indicating poor quality; 4–5, moderate quality; and 6+, good quality. Among our 51 included studies, 30 were classified as good quality, 19 as moderate quality, and 2 as low quality (Supplementary Table 5).

## Results

### Overview of study characteristics

**Fig. 1:**
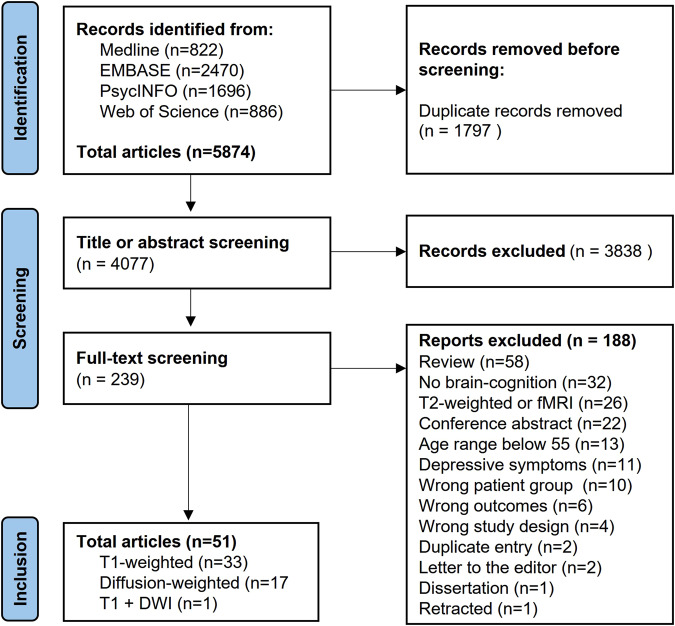
PRISMA flow chart illustrating the number of records included and excluded at various screening stages, leading to final list of records for inclusion and data extraction. Abbreviations: DWI: diffusion-weighted imaging; fMRI: functional magnetic resonance imaging; T1: T1-weighted imaging.

Our search identified 4,077 eligible studies after the removal of duplicates. After title and abstract screening, 3,838 were excluded and 239 progressed to full-text screening with 188 studies excluded at this stage, yielding 51 studies with 33 studies using T1 imaging, 17 using DWI, and 1 using both (Fig. 1). Table 1 presents the characteristics of the T1 longitudinal studies and Table 2 presents the T1 cross-sectional studies, including the way data were analyzed: region of interest (ROI), voxel-based morphometry (VBM), or structural network analysis. Table 3 presents the characteristics of the DWI studies, including the way data were analyzed: ROI or voxel-wise analysis.

**Table 1:** Brain-cognition associations in magnetic resonance imaging (MRI) studies of late-life depression (LLD): T1 longitudinal findings.

#	Reference	Study Design	N LLD/HC (EOD/LOD)	Depression Status	Methods to Analyze Imaging Data	Neuropsychological Assessment	Summary of Significant Findings
**1**	Kohler et al. (2010)	longitudinal (18-month follow-up)	57 35/29	current	ROI (subcortical volumes) HIPP	**Episodic memory**^**b**^ (RAVLT: immediate recall, delayed recall, and delayed recognition) **Executive function**^**b**^ (The FAS verbal fluency test, TMT A/B, SCWT) **Processing speed**^**b**^ (VIGIL)	**LLD** No significant associations
**2**	Hou et al. (2011)	longitudinal (21-month follow-up)	33 14/19 (0/14)	remitted	ROI (subcortical volumes) HIPP (CA1, CA2, CA3, CA4 subfields, subiculum, alveus, and fimbria)	MMSE **Verbal memory** (RAVLT delayed recall) **Executive function** (TMT A and B, DST) **Attention and processing speed** (SDMT)	**LLD** Decrease in R. HIPP volume associated with decrease in SDMT score. Decrease in L. HIPP volume associated with decrease in MMSE score. **HC** No significant associations
**3**	Sachs-Erisson et al. (2011)	cross-sectional & longitudinal (4-year follow-up)	61 61/0	current	ROI (subcortical volumes) HIPP	MMSE	**LLD** *Longitudinal findings* Smaller HIPP at baseline predicted decreased MMSE scores over 4 years. **Other** Smaller HIPP at baseline plus APOE E4 allele associated with more cognitive decline over 4 years
**4**	Steffens et al. (2011)	longitudinal (2.5-year follow-up)	162 90/72	current	ROI (subcortical volumes) HIPP	MMSE	**LLD** Decrease HIPP volume associated with decreased MMSE scores over 2–2.5 years. **HC** No significant association
**5**	Sawyer et al. (2012)	Longitudinal (up to 10-year follow-up)	384 238/146	current	ROI (subcortical volumes) HIPP	MMSE	Smaller HIPP at baseline predicted decreased MMSE scores over 4 years
**6**	Marano et al. (2015)	clinical trial (12 weeks; citalopram)	34 17/17	current	VBM	**Verbal memory** (CVLT) **Executive function** (letter fluency of DKEFS)	**LLD** *Executive function* Smaller R. superior frontal gyrus, R. middle frontal gyrus, L. fusiform gyrus, and L. cerebellum at baseline associated with less improvement in DKEFS scores. Smaller precuneus volume at baseline associated with more improvement in DKEFS scores^a^ *Verbal memory* Smaller L. inferior frontal gyrus, R. superior temporal gyrus, R. uncus, bilateral fusiform, R. angular gyrus, and R. lingual gyrus at baseline associated with less improvement in CVLT score. Smaller superior frontal gyrus at baseline associated with more improvement in CVLT score
**7**	Droppa et al. (2017)	clinical trial (12 week; venlafaxine)	26 26/0	current	ROI (cortical and subcortical volumes) AMY, HIPP, parahippocampus, inferior, middle, and superior frontal gyrus (operculum, triangular, and orbital)	**Processing speed** (coding task, RBANS) **Delayed verbal memory** (list and story recall, RBANS) **Delayed visuospatial memory** (figure recall, RBANS) **Set shifting** (TMT, DKEFS) **Response inhibition** (CWI) **Inhibition and set shifting** (CWI: inhibition component)	**LLD** No significant associations

^a^Unexpected finding (i.e., reduced gray matter volume or thickness associated with better neuropsychological performance)

^b^Cognitive domain composite

*AMY* amygdala, *APOE* Apolipoprotein E, *CA* cornu ammonis, *CVLT* California Verbal Learning Test, *CWI* Color Word Interference, *DST* Digit Span Test, *EOD* early-onset depression, *FAS* Controlled Oral Word Association Test, *HC* healthy controls, *HIPP* hippocampus, *L* left, *LOD* late-onset depression, *MMSE* mini mental state examination, *LLD* late-life depression, *R* right, *RAVLT* The Rey Auditory Verbal Learning Test, *ROI* region of interest, *SCWT* The Stroop Color Word Test, *SDMT* Symbol Digit Modalities Test, *SUP* superior, *TMT* Trail-making Test, *VBM* voxel-based morphometry, *VIGIL* computerized continuous performance task

**Table 2:** Brain-cognition associations in magnetic resonance imaging (MRI) studies of late-life depression (LLD): T1 cross-sectional findings.

#	Reference	Study Design	N LLD/HC (EOD/LOD) [Other]	Depression Status	Methods to Analyze Imaging Data	Neuropsychological Assessment	Summary of Significant Findings
1	Greenwald et al. (1997)	cross-sectional	66 30/36	current	ROI (cortical and subcortical atrophy)	Extended MMSE	**LLD** Medial temporal and third ventricles size associated with lower extended MMSE scores. Right ventricle size associated with lower MMSE scores. **HC** No significant associations
2	Pantel et al. (1997)	cross-sectional	32 19/13 (0/19) [AD: 27]	current	ROI (cortical and subcortical volumes) HIPP/AMY complex Frontal lobes Temporal lobes	MMSE	**LLD** No significant associations **AD** Smaller HIPP/AMY complex volume associated with lower MMSE scores in the AD group
3	Dahabra et al. (1998)	cross-sectional	17 17/0 (9/8)	remitted	ROI (cortical volumes) Temporal lobe and ventricle volume	MMSE **Verbal memory** (Warrington verbal recognition test, Story recall (immediate and delayed) by the Coughlan Test) **Visual memory** (Spatial recognition, sample matching, and conditional associative learning of the CANTAB)	**LLD** No significant associations
4	Ashtari et al. (1999)	cross-sectional	86 40/46 (16/24)	current	ROI (cortical and subcortical volumes) HIPP formation ANT-HIPP/AMY complex Whole brain	MMSE	**LLD** Smaller volumes of R. HIPP formation, L. HIPP formation, R+L HIPP formation, and HIPP/AMY complex volumes associated with lower MMSE scores. **HC** No significant associations
5	Lai et al. (2000)	cross-sectional	40 20/20	current	ROI (cortical volumes) OFC	MMSE	**LLD or HC** No significant associations
6	Steffens et al. (2000)	cross-sectional	84 66/18 (28/38)	current	ROI (subcortical volumes) HIPP	MMSE	**LLD** Smaller L. HIPP volume associated with lower MMSE scores. **HC** No significant associations **EOD, LOD** Significant brain-cognition associations in LOD, but not EOD
7	Bell-McGinty et al. (2002)	cross-sectional	77 30/47	current	Voxel wise	MMSE	**LLD** No significant associations
8	Almeida et al. (2003)	cross-sectional	88 51/37 (24/27)	current	ROI (cortical volumes) R. frontal L. frontal whole brain	CAMCOG	**LLD+HC** Smaller R. frontal, L. frontal, and whole brain associated with lower CAMCOG scores. **EOD, LOD** Significant associations among EOD and HC, not LOD
9	Steffens et al. (2003)	cross-sectional	70 30/40	current	ROI (subcortical volumes) OFC	MMSE **Visual memory** (BVRT—perseverative errors and number correct)	**LLD or LLD+HC** Smaller L. OFC associated with worse visual memory
10	Lloyd et al. (2004)	cross-sectional	80 51/39 (23/28)	current	ROI (subcortical volumes) HIPP	CAMCOG MMSE	**LLD or HC** No significant associations
11	Yuan et al. (2008)	cross-sectional	35 19/16 (0/19)	remitted	VBM	**Verbal memory** (RAVLT Delayed Recall). **Executive function** (TMT A and B, DST) **Visuospatial skills** (CDT)	**LLD** Smaller L. cingulate gyrus associated with better episodic memory^a^
12	Ballmaier et al. (2008)	cross-sectional	43 28/15 (15/13)	current	ROI (subcortical volumes) HIPP	MMSE **Verbal learning**: CVLT **Visual memory**: ROCF	**LLD** Smaller R. HIPP associated with worse memory. **EOD, LOD** Brain-cognition association significant among in LOD, not EOD
13	Egger et al. (2008)	cross-sectional	34 14/20 (0/14)	current	VBM (subcortical volumes)	MMSE **Verbal memory** (CERAD) **Learning** (CERAD) **Free recall and recognition** (CERAD) **Figural memory** (free recall, CERAD) **Object naming** (BNT—short version, CERAD) **Verbal fluency** (animals/min, CERAD) **Planning** (CLOX 1) **Constructive ability** (copy geometrical shapes, CERAD; ClOX 2) **Divided attention** (TMT B)	**LLD+HC** Smaller HIPP, PUT, nucleus caudate, L. thalamus, and insula associated with worse performance on “CERAD constructional praxis”
14	Sheline et al. (2008)	cross-sectional	115 83/32	current	Whole-brain volumetric analysis	**Language processing**^**b**^ (Shipley Vocabulary Test, BNT, word reading condition of the Stroop) **Processing speed**^**b**^ (Symbol-digit modality, color naming condition of the Stroop, TMT A) **Working memory**^**b**^ (Digit span forward, digit span backward, ascending digits) **Episodic memory**^**b**^ (Word list learning, logical memory, CP20, BVRT) **Executive function**^**b**^ (Verbal fluency, TMT B, Stroop CWI21, I/P subscales of the Mattis DRS)	**LLD** Smaller whole brain associated with slower processing speed. **HC** No significant associations
15	Elderkin -Thompsn et al. (2009)	cross-sectional	49 26/23	current	ROI (cortical volumes) ANT CING OFC Gyrus rectus	MMSE **Executive function** (WAIS-III) **Visuoconstruction** (block design) **Nonverbal inductive reasoning** (matrix reasoning) **Manipulation and sequencing of information** (letter-number sequences) **Nonverbal fluency** (Ruff Figural Designs) **Verbal fluency** (FAS)	**LLD+HC** Smaller ANT CING or gyrus rectus associated with worse executive function (Block Design, Letter-Number Sequences, and Matrix Reasoning). Smaller OFC associated with better verbal and nonverbal memory^a^
16	Yuan et al. (2010)	cross-sectional	37 37/0 (0/37)	remitted	VBM	MMSE **Verbal memory** (RAVLT) **Executive function** (TMT A and B, DST) **Visuospatial skills** (CDT, ROCF) **Attention and processing speed** (SDMT)	**LLD** No significant associations **ApoE e4 carriers** Smaller R. medial frontal gyrus associated with lower Digit Span Test score in ApoE e4 carriers
17	Avila et al. (2011)	cross-sectional	79 48/31 (20/28)	current	VBM (cortical and subcortical volumes)	MMSE **Verbal episodic memory** (FOME, Wechsler Memory Scale-Revised Logical Memory I and II) **Visual episodic memory** (Wechsler Memory Scale Revised Visual Reproduction I and II) **Executive function** (FAS test, Stroop Test, TMT B) **Processing speed** (Stroop A and B, TMT A)	**LLD** Smaller L. HIPP and bilateral parahippocampal gyrus associated with worse verbal episodic memory Smaller L. parahippocampal gyrus associated with worse verbal and visual episodic memory **HC**Smaller R. HIPP associated with lower MMSE scores Smaller bilateral parahippocampal gyrus associated with worse verbal episodic
18	Chang et al. (2011)	cross-sectional	123 88/35	current	ROI (subcortical volumes) DLPFC (SFG and MFG)	MMSE	**LLD** Smaller L. DLPFC associated with lower MMSE scores
19	Colloby et al. (2011)	cross-sectional	68 38/30	current and remitted (mix)	ROI (thickness) VBM DARTEL (volume)	MMSE	No significant associations
20	Sachs-Erisson et al. (2011)	cross-sectional & longitudinal (4-year follow-up)	61 61/0	current	ROI (subcortical volumes) HIPP	MMSE	**LLD** *Cross-sectional findings* No significant associations **Other** Smaller HIPP at baseline plus APOE E4 allele associated with more cognitive decline over 4 years
21	Lamar et al. (2012)	cross-sectional	51 18/33	current	*ROI (cortical volumes) PFC, CING cortex, temporal cortex, parietal cortex	**Verbal episodic memory** (CVLT - immediate recall and delayed recall, Logical Memory subtest from the WMS III for story-based recall)	**LLD** Smaller PFC region (driven by OFC) and R. cingulate cortex (driven by ANT CING) associated with lower verbal episodic memory. **HC** No significant associations
22	Lim et al. (2012)	cross-sectional	95 48/47 (0/48)	current	ROI (cortical thickness; subcortical volumes)	MMSE **Language** (verbal fluency, BNT) **Verbal memory** (WLM, WLR, WLRc) **Visual memory** (CP, CR) **Executive function** (Stroop CWI)	**LLD** *Cortical thickness* Thinner PCUN, insula, L. superior temporal, R. inferior temporal associated with lower verbal learning (immediate recall). Thinner L. FUS, L. ENTOR, insula, PCUN, L. PCEN, R. isthmus CING, R. SMG, R. inferior parietal associated with lower verbal learning (delayed recall). Thicker R. DLPFC, R. superior frontal, R. PCEN, R. PCUN, R. medial orbitofrontal, R. rostral ANT CING, R. rostral middle frontal area, L. insula associated with worse executive functiona *Subcortical volumes* Smaller R. PUT and HIPP associated with lower verbal fluency
23	Sexton et al. (2012)^c^	cross-sectional	61 36/25	remitted	ROI (cortical and subcortical volumes) Whole-brain volume and HIPP volume	**Executive function**^**b**^ (digit span forward and backward, letter fluency, TMT B) **Processing speed**^**b**^ (digit symbol, TMT A, CANTAB: simple reaction time, simple movement time, five-choice reaction time, five-choice movement time). **Episodic memory**^**b**^ (visual episodic memory; ROCF immediate and delay, verbal episodic memory; HVLT: total, recall, and recognition) **Language skills**^**b**^ (graded naming test, category fluency) **Visuospatial skills**^**b**^ (CDT, copied drawings, and RCF: copy)	**LLD** Smaller whole brain and R. HIPP associated with lower episodic memory function.
24	Xie et al. (2012)	cross-sectional	72 18/25 [aMCI: 17 LLD+aMCI: 12]	current	Voxel wise	MMSE **Episodic memory** (logical memory II delayed paragraph recall from the Wechsler memory scale)	**LLD, LLD+aMCI, MCI, or HC** No significant brain-cognition associations in any single groups **LLD, LLD+aMCI, MCI, and HC** No significant associations with episodic memory. The interaction of lower episodic memory with higher depressive symptoms was associated with smaller R. anterior insula/inferior frontal gyrus and L. medial frontal gyrus.
25	Lebedeva et al. (2015)	cross-sectional	98 49/49 (25/24)	current	ROI (subcortical volumes) HIPP Vertex-wise (cortical thickness)	MMSE **Verbal Memory** (CERAD-10 Word Test, Word Fluency Test)	**LLD** Smaller R. HIPP is associated with lower MMSE scores. Thinner superior frontal gyrus associated with lower MMSE scores. **Other** Brain-cognition associations significant in LOD, but not EOD
26	Jayaweera et al. (2016)	cross-sectional	111 84/27 (50/34)	current + remitted	ROI (subcortical volumes) ANT/POST subregions of the caudate nucleus, HIPP	MMSE **Verbal episodic memory** (RAVLT, logical memory of the WMS-III)	**LLD** Smaller R. anterior caudate associated with worse verbal memory **HC** No significant associations
27	Choi et al. (2016)	cross-sectional	100 50/50 (0/50)	current	ROI (subcortical volumes) HIPP subfields (CA1, CA2, CA3, CA4, DG, SUB)	MMSE **Language** (verbal fluency, 15-item BNT) **Verbal memory** (WLM, WLR, WLRc) **Visual memory** (CP, CR) **Executive function** (Stroop CWI)	**LLD** Smaller L. CA1 and total HIPP associated with worse verbal episodic memory.
28	Shin et al. (2018)	cross-sectional	98 50/48	current	Structural Network Analysis (cortical thickness)	MMSE **Language** (Verbal fluency, 15-item BNT) **Verbal memory** (WLM, WLR, WLRc) **Visual memory** (CP, CR) **Executive function** (Stroop CWI)	**LLD** Thinner cortex in a sub-network of 14 brain regions (core regions: right middle anterior-cingulate cortex; right posterior transverse collateral sulcus) associated with worse executive function A sub-network of 36 brain regions with cores belonging to the left subcentral cortex, right precuneus, and the posterior ramus of the right lateral sulcus was correlated with verbal fluency scores. Thinner cortex in fusiform and lateral occipital gyrus associated with worse verbal memory.

*Composite brain variables were used for each of the listed regions. PFC (orbitofrontal cortex (medial and lateral) inferior frontal gyrus (pars opercularis, pars triangularis and pars orbitalis) rostral division of the middle frontal gyrus), CING cortex (rostral anterior caudal anterior posterior divisions), temporal cortex (entorhinal cortex, parahippocampal gyrus, middle temporal gyrus), parietal cortex (superior parietal, inferior parietal, precuneus cortices)

^a^Unexpected finding (i.e., lower gray matter volume or thickness associated with better neuropsychological performance)

^b^Cognitive domain composite

^c^Unique study reporting on both T1 and diffusion weighted imaging (DWI) findings

*AD* Alzheimer’s disease, *aMCI* amnestic mild cognitive impairment, *AMY* amygdala, *ANT* anterior (ANT), *APOE* Apolipoprotein E, *BNT* Boston Naming Test, *BVRT* Benton Visual Retention, *CA* cornu ammonis, *CAMCOG* Cambridge Cognition Examination, *CANTAB* Cambridge Neuropsychological Test Automated Battery, *CERAD* Consortium to Establish a Registry for Alzheimer’s Disease, *CING* cingulate, *CLOX* clock drawing task, *CP* Constructional Praxis, *CR* Constructional Recall, *CVLT* California Verbal Learning Test, *CWI* Color Word Interference, *DG* dentate gyrus, *DLPFC* dorsolateral prefrontal cortex, *DRS* Dementia Rating Scale, *DST* Digit Span Test, *ENTOR* entorhinal, *EOD* early-onset depression, *FAS* Controlled Oral Word Association Test, *FOME* Fuld Object-Memory Evaluation, *FUS* fusiform, *HIPP* hippocampus, *HVLT* Hopkins Verbal Learning Test, *I/P* Initiation/Perseveration, *L* left, *LLD* late-life depression, *LOD* late-onset depression, *MFG* middle frontal gyrus, *MMSE* mini mental state examination, *OFC* orbitofrontal cortex, *PCEN* precentral, *PCUN* precuneus, *PFC* prefrontal cortex, *POST* posterior, *PUT* putamen, *R* right, *RAVLT* The Rey Auditory Verbal Learning Test, *ROCF* Rey–Osterrieth Complex Figure, *ROI* region of interest, *SDMT* Symbol Digit Modalities Test, *SFG* superior frontal gyrus, *SMG* supramarginal, *SUB* subiculum, *TMT* Trail-making Test, *VBM* voxel-based morphometry, *WLM* Word List Memory, *WLR* Word List Recall, *WLRc* Word List Recognition

**Table 3:** Brain-cognition associations in magnetic resonance imaging (MRI) studies of late-life depression (LLD): diffusion-weighted imaging (DWI).

#	Reference	Study Design	N LLD/HC (EOD/LOD) [Other]	Depression Status	Methods to Analyze Imaging Data	Neuropsychological Assessment	Summary of Significant Findings
1	Alexopoulos et al. (2002)	clinical trial (12 weeks; citalopram)	13 13/0	current	ROI (FA) A priori ROI: AC-PC Plane ROI 15 and 10 mm above the AC-PC plane	**Executive function** (I/P domain of Mattis DRS, Stroop CWI)	**LLD** ↓ FA of regions 15 (R.) and 10 mm (bilateral) above the AC-PC plane associated with worse performance on Stroop CWI
2	Murphy et al. (2007)	cross-sectional	51 51/0	current	Voxel-wise (FA)	Stroop CWI	**LLD** ↓ FA of white matter lateral to the ANT/POST CING cortex, L. prefrontal, insular, posterior temporal, parahippocampal, and occipital regions associated with worse performance on Stroop CWI
3	Yuan et al. (2007)	cross-sectional	30 16/14 (0/16)	remitted	Voxel-wise (FA)	**Verbal memory** (RAVLT Delayed Recall). **Executive function** (TMT A and B, DST) **Visuospatial skills** (CDT)	**LLD** ↓ FA of R. superior frontal gyrus associated with worse performance on TMT B
4	Shimony et al. (2009)	cross-sectional	96 73/23	current	ROI (MD, RD) ^b^A priori ROI listed below.	**Language**^a^ (Shipley Vocabulary test, BNT, and the word reading condition of the Stroop) **Processing speed**^a^ (SDM, color naming of the Stroop task, and TMT A) **Working memory**^a^ (digit span forward and backward, and ascending digits) **Episodic memory**^a^ (word list learning, logical memory, CP, and the BVRT) **Executive function**^a^ (verbal fluency, TMT B, Stroop CWI Test, I/P domain of the Mattis DRS, and categories completed from the Wisconsin Card sorting test)	**LLD** ↑ MD and ↓ RA of prefrontal region associated with slower processing speed ↑ MD of deep white matter ROIs and CC associated with slower processing speed **HC** No significant associations
5	Yuan et al. (2010)	cross-sectional	70 37/33 (0/37)	remitted	ROI (FA) A priori ROI: IFOF (frontal, frontotemporal, temporal) Genu and splenium of ANT and POST CC CING bundle (mid and POST CING) SLF II	**Verbal memory** (RAVLT) **Executive function** (TMT A and B, DST) **Visuospatial skills** (CDT, ROCF) **Attention and processing speed** (SDMT)	**LLD** ↓ FA of L. posterior cingulate bundle associated with worse performance on TMT A **HC** No significant associations
6	Alves et al. (2012)	cross-sectional	40 17/18	current	TBSS (FA)	CERAD Tests: **Verbal fluency** (semantic category) **CP** (figure copying) **Language** (BNT) **Episodic memory** (word list learning, delayed free recall, word recognition) **Processing speed** (TMT A) **Executive function** (TMT B)	**LLD+HC** ↓ FA of R. posterior cingulate cluster associated with worse verbal fluency, immediate word recall, and delayed recall **LLD** ↓ FA of R. posterior cingulate cluster is associated with worse language skill. **HC**No significant associations
7	Mettenburg et al. (2012)	cross-sectional	67 51/16	remitted and current	TBSS (FA, MD, AD, RD) A priori ROI*:* CING, UF, CC (genu, body, splenium)	*See Shimony et al. (2009)*	**LLD+HC** *Fractional anisotropy* ↓ FA of L. CING associated with worse language ↓ FA of R. CING associated with worse language and episodic memory ↓ FA of L. UF associated with worse executive function and language ↓ FA of R. UF associated with worse episodic memory *Radial diffusivity* ↑ RD of L. CING associated with worse executive function ↑ RD of R. CING associated with worse executive function and slower processing speed ↑ RD of L. UF associated with worse executive function, language, and working memory ↑ RD of R. UF associated with worse executive function
8	Sexton et al. (2012)^c^	cross-sectional	61 36/25	remitted	TBSS (FA)	**Executive function**^a^ (digit span forward and backward, letter fluency, TMT B) **Processing speed**^a^ (DST, TMT A, CANTAB: simple reaction time, simple movement time, five-choice reaction time, five-choice movement time). **Episodic memory**^a^ (visual episodic memory; ROCF: immediate and delay, verbal episodic memory; HVLT: total, recall, and recognition) **Language skills**^a^ (graded naming test, category fluency) **Visuospatial skills**^a^ (CDT, copied drawings, and ROCF)	**LLD** ↓ FA of ANT TR and UF associated with worse executive function ↓ FA of CC (genu) is associated with worse processing speed ↓ FA of the anterior TR, CC (body, genu, and fornix associated with worse episodic memory).
9	Lamar et al. (2013)	cross-sectional	60 26/34	current	TBSS and ROI Deterministic Tractography (FA, AD, RD) A priori ROI: UF and CING	MMSE **Executive function** (SOPT, OA) **Episodic memory**^a^ (Immediate and long delay free recall of the CVLT, Immediate and delay free recall (WMS-III), Logical Memory I and II (WMS-III), Visual Reproduction I and III from the (WMS-III))	*TBSS* **LLD** No significant associations **HC** ↑ RD of R. internal capsule, L. POST corona radiata, L. POST TR, inferior IFOF, ILF, and SLF associated with worse executive function (SOPT). ↓ FA of CC (genu), ANT TR, ANT corona radiata, UF, CING, and IFOF associated with worse executive function (OAE/T). ↑ RD of CC (genu), R. splenium, ANT corona radiata, CING, ANT TR, IFOF, R. UF, and L. ILF associated with worse executive function (OAE/T) *Deterministic Tractography* **LLD** ↓ FA of R. UF associated with worse executive function (OAE/T) **HC** ↓ FA of R. CING associated with worse executive function (SOPT9)
10	Li et al. (2014)	cross-sectional	84 20/33 [aMCI:18 aMCI+LLD:13]	current	TBSS (FA, MD, AD, RD) A priori ROI: CING CING-HIPP CC UF Fornix	MMSE **Episodic memory** (logical memory II delayed paragraph recall subscale from the WMS)	**LLD, aMCI, aMCI+LLD, or HC** No significant brain-cognition associations in any groups **LLD, aMCI, aMCI+LLD, and HC** ↑ MD and ↑ RD of CING-HIPP tract associated with worse episodic memory in whole sample
11	Charlton et al. (2014)	cross-sectional	46 23/23	current	ROI - Tractography (FA, MD, AD, RD) A Priori ROI: UF and CING	**Learning and memory**^a^ (CVLT-II, Logical Memory I and II (WMS-III), and Visual Reproduction I and II (WMS-III)) **Executive function**^a^ (Category switching total correct from the DKEFS, TMT B, Stroop CWI, Backwards DST, SOPT total errors)	**LLD+HC** No significant associations **LLD** No significant associations **HC** ↑ MD and RD of L. UF associated with worse learning and memory; ↑ MD, AD, and RD of R. UF associated with worse learning and memory ↑ MD, AD, and RD of L. CING associated with worse learning and memory; ↑ MD and AD of R. CING associated with worse learning and memory ↑ MD, AD, and RD of R. UF associated with worse executive function performance; ↑ AD of L. CING associated with worse executive function performance
12	Yin et al. (2016)	cross-sectional	71 32/39 (0/32)	current	ROI -deterministic tractography (FA, MD, AD, RD) A priori ROI: Tracts connecting PCC/Pcu with dACC Tracts connecting PCC/Pcu with thalamus	**Episodic memory** (AVLT) **Working memory** (DST) **Semantic memory** (VFT) **Perceptual speed** (TMT-A) **Executive function**(TMT-B, and SDMT).	**LLD+HC** ↓ FA and ↑ RD of tracts between PCC/Pcu and dACC associated with worse working memory and executive function
13	Li et al. (2017)	longitudinal (baseline cross-sectional brain-cognition analysis)	48 24/24	remitted	Probabilistic tractography; NBS	**Executive Function**^a^ **(**TMT B, Stroop CWI) **Memory**^a^ **(**Rivermead immediate and delay Story Recall, HVLT-R Immediate and Delayed Story Recall) **Processing speed**^a^ (Digit Span (WAIS-III), DSST(WAIS-III))	**LLD** ↓ connectivity in subnetwork (L. lingual gyrus, L. middle occipital gyrus, and L. fusiform gyrus) associated with worse processing speed
14	Mai et al. (2017)	cross-sectional	69 24/30 (0/39) [“LLD with memory deficit”: 15]	current	Deterministic tractography; NBS	**Executive function**^a^ **(**TMT A/B, Stroop Color and Word Test, DST) **Processing speed**^a^ **(**Stroop A, TMT A, SDMT) **Memory**^a^ **(**AVLT, LMT)	**LLD** ↓ connectivity in widespread network (frontal, temporal, parietal, limbic, and subcortical regions) associated with worse executive function, processing speed, and memory. **HC** No significant associations
15	Wang et al. (2020)	cross-sectional	67 37/30 (0/37)	remitted	ROI (FA, MD) ROI: ICBM-DTI-81 Atlas	**Delayed verbal memory** (RAVLT) **Executive function** (TMT A and B, DST) **Visuospatial skills** (CDT, ROCF, ROCF - delayed recall) **Attention and processing speed** (SDMT)	**LLD** No significant associations
16	He et al. (2021)	clinical trial (8 weeks; escitalopram or duloxetine)	71 71/0	current	TBSS (FA)	**Executive function** (Stroop CWI Test, I/P domain (DRS)) **Processing speed** (DST from the WAIS-III) **Episodic memory** (logical memory component of the WMS-R)	**LLD** *Executive function* ↓ FA in SLF, SLF-temporal, and the R. CST associated with worse executive function (I/P) ↓ RD in ANT TR, CST, R. UF, R. SLF, R. SLF-temporal, R. IFOF, R. ILF, and R. forceps major associated with improvement in executive function (I/P)
17	Wang et al. (2021)	cross-sectional	76 40/36 (0/40)	remitted	ROI (FA, MD)	**Episodic memory**^**a**^ (AVLT-DR and ROCF-DR) **Visuospatial function**^**a**^ (ROCF and CDT) **Processing speed**^**a**^ (SDMT and TMT-A) **Executive function**^**a**^ (DST and TMT-B).	**LLD** ↓ FA (L. CST and ANT corona radiata) and ↑ MD (fornix, R. cerebral peduncle, R. EC, R. PCC and R. UF) predicted worse processing speed.
18	Zhou et al. (2022)	Cross-sectional	142 74/68	current	ROI (FA)	**Memory**^**a**^ (ROCF-Delay Recall test And RAVLT) **Executive Function**^**a**^ (TMT-B and Stroop CWI) **Attention**^**a**^ (TMT-A and SDMT) **Language** (Verbal Fluency Test and BNT) **Visuospatial Ability**^**a**^ (Clock Drawing Test 4 and ROCF-Copy) **Global Cognition** (MMSE)	**LLD** *Global Cognition* ↓ FA in the L. CING and forceps major associated with worse global cognition (MMSE) *Executive Function* ↓ FA in R. and L. ANT TR associated with worse executive function. **HC** No significant associations

^a^Cognitive domain composites.

^b^Regions selected: Frontal regions (superior frontal gyrus, middle frontal gyrus, inferior frontal gyrus, medial orbital frontal, lateral orbital frontal, dorsal cingulate, anterior cingulate, ventral cingulate, mesial fronto-polar cortex); Temporal regions (medial temporal gyrus, fusiform gyrus, auditory cortex); Parietal regions (somatosensory cortex, posterolateral intra parietal sulcus); Occipital regions (occipital pole, visual cortex)

^c^Unique study reporting on both T1 and diffusion weighted imaging (DWI) findings

*AC-PC* anterior commissure–posterior commissure, *AD* axial diffusivity, *aMCI* amnesic mild cognitive impairment, *ANT* anterior, *BNT* Boston Naming Test, *BVRT* Benton Visual Retention Test, *CANTAB* Cambridge Neuropsychological Test Automated Battery, *CC* corpus callosum, *CERAD* Consortium to Establish a Registry for Alzheimer’s Disease, *CING* cingulate bundle, *CING-HIPP* cingulum-hippocampus, *CP* Constructional Praxis, *CST* corticospinal tract, *CVLT* California Verbal Learning Test, *CWI* Color Word Interference, *dACC* dorsal anterior cingulate cortex, *DKEFS* The Delis-Kaplan Executive Function System, *DR* delayed recall, *EC* external capsule, *DRS* Dementia Rating Scale, *DSST* Digit–Symbol Substitution Test, *DST* digit-span test, *FA* fractional anisotropy, *HVLT* Hopkins Verbal Learning Test, *I/P* initiation/Perseveration, *ICBM-DTI-81* International Consortium of Brain Mapping probabilistic white matter atlas, *IFOF* inferior fronto-occipital fasciculus, *ILF* inferior longitudinal fasciculus, *L* left, *LMT* Logical Memory Test, *MD* mean diffusivity, *MMSE* mini mental state examination, *NBS* network-based statistic, *OA* Investigations of the Object Alternation Task, *PCC* posterior cingulate cortex, *Pcu* precuneus, *POST* posterior; *R* right, *RAVLT* The Rey Auditory Verbal Learning Test, *RD* radial diffusivity, *ROCF* Rey–Osterrieth Complex Figure, *ROI* region of interest, *SDM* symbol-digit modality, *SDMT* Symbol Digit Modalities Test, *SLF* superior longitudinal fasciculus, *SOPT* The Self-Ordered Pointing Task, *TMT* Trail-making Test, *CDT* clock-drawing test, *TR* thalamic radiation, *UF* uncinate fasciculus, *VFT* verbal fluency test, *WAIS* The Wechsler Adult Intelligence Scale, *WMS* The Wechsler Memory Scale

Of the 51 studies, 41 compared LLD and HC groups (Supplementary Table 5); 7 included only an LLD group (Supplementary Table 6); and 3 studies included additional comparison groups: two studies compared patients with LLD, MCI, or LLD + MCI, and HC and one study compared patients with LLD or LLD plus a memory deficit (not meeting criteria for MCI), and HC (Supplementary Table 7). Thirty-eight studies included only patients during a current depressive episode, 10 studies included only patients with remitted depression, and 3 included patients with current or remitted depression. Ten of the 51 studies also compared findings in patients with EOD versus LOD, and 9 included only patients with LOD.

### T1 studies

#### T1 longitudinal studies

Of the 33 T1 studies, 7 used a longitudinal design in participants with LLD and an HC group or LLD only [26–32]. Four of these studies reported significant positive associations between the bilateral hippocampal volumes and changes in the Mini-Mental State Examination (MMSE) scores during follow-up periods lasting from 21 months to 4 years [27, 30–32]. This association was not present in the HC group [27, 32].

These studies also evaluated the relationship between GM volumes and specific cognitive domains including tests of processing speed [27], visuospatial memory [26], response inhibition and set-shifting [26], and verbal fluency and memory [29]. Hou et al. (2012) showed that larger right hippocampal volume predicted improved performance on the Symbol Digit Modalities Test over a 21-month period [27]. No significant longitudinal associations between baseline hippocampal volume and persistent cognitive decline in episodic memory, executive function, or processing speed were reported over 18 months [28].

##### T1 treatment studies

Two studies explored pre- and post-treatment associations between GM volume and cognition during a treatment trial [26, 29]. In the first trial, 17 participants with LLD were treated with citalopram [29]. In this voxel-based morphometry (VBM) analysis, larger baseline gray matter volumes of frontal regions (including the right superior and middle frontal gyri) and the left fusiform gyrus were associated with improvement in verbal fluency following treatment [29]. Larger baseline volumes of the left middle frontal gyrus, left inferior frontal gyrus, right superior temporal gyrus, right uncus, bilateral fusiform gyrus, right angular gyrus, and right lingual gyrus were also associated with improvement in verbal memory following treatment [29]. Interestingly, smaller baseline gray matter volume of the bilateral precuneus and superior frontal gyrus were associated with improved verbal fluency and verbal memory, respectively, following treatment [29]. The second trial of 26 participants treated with venlafaxine did not find significant associations between longitudinal changes in brain structure and cognition following treatment [26].

#### T1 cross-sectional studies

##### Global cognition: MMSE or CAMCOG only

Twelve cross-sectional studies assessed the relationship between GM volumes or thickness and global cognition measured by the MMSE. Of these 12 studies, 4 reported significant positive associations with medial temporal lobe (MTL) region volumes including the hippocampus and hippocampal-amygdala complex in LLD [33–36], but not in HC [33, 36]. In two studies using an ROI analysis in patients with LLD, GM volume of the left DLPFC was positively associated with MMSE scores and left/right frontal lobe volume was positively associated with the Cambridge Cognition Examination (CAMCOG) scores [37, 38]. The latter finding was also significant in the HC group [37]. A vertex-wise analysis of cortical thickness in an LLD group also reported a positive association between the thickness of the superior frontal gyrus bilaterally and MMSE scores [35]. Finally, 6 of the 12 studies reported no associations between any GM measure and impaired global cognition [30, 39–43]. Only half of the cross-sectional studies assessing the relationship between GM volumes or thickness and global cognition reported associations.

##### Domain-specific cognition

**Executive functioning and processing speed**

Six studies used a detailed cognitive battery to test for brain-cognition associations with executive dysfunction and slower information processing speed in LLD. Two of these 6 studies used a whole-brain approach and reported that lower cortical thickness of frontal-executive (e.g., DLPFC, rostral middle frontal) and corticolimbic regions (e.g., superior prefrontal and frontal cortices, anterior cingulate, precuneus) was significantly associated with executive dysfunction in LLD [44, 45]. Of note, Lim et al. (2012) LLD group included only patients with LOD. Another study using an ROI approach identified an association between lower GM volume in the corticolimbic region, including the gyrus rectus and the orbitofrontal cortex (OFC), with executive dysfunction. [46]. The association between the gyrus rectus volume loss and executive dysfunction differed significantly between the LLD and HC groups, with a weaker association in the LLD group [46]. An association of lower cortical thickness in the superior temporal gyrus with executive dysfunction was also identified in the whole-brain analyses by Shin et al. (2018). There were no correlations between any measure of subcortical volumes and executive functioning [44]. Finally, one study found significant associations between lower whole-brain GM volume and slower processing speed in LLD, but not in HC [47] . However, no associations between whole brain GM volumes using VBM and executive function were reported by Yuan et al. (2008) [48]. Generally, cross-sectional T1 studies of brain-cognition relationships implicate different frontal and temporal regions.

**Learning and episodic, verbal, or visuospatial memory**

Fifteen studies examined associations between GM volumes or thickness and cognitive performance on tests of learning and episodic, verbal, or visuospatial memory. Of those, eight used an ROI approach, six conducted whole-brain voxel-wise or vertex-wise analyses, and one conducted a structural network analysis. The evidence strongly supports the role of MTL regions in learning and memory in LLD: 3 of the 8 ROI studies [7, 49, 50] and 3 of the 6 voxel-wise studies [44, 51, 52] found significant positive correlations between smaller bilateral hippocampal volume and lower scores of tests assessing episodic, verbal, and visual memory. These associations were also significant in remitted LLD [7]. One study also implicated the parahippocampal gyrus in performance on immediate verbal memory tests [51]. Two ROI studies implicated the OFC in verbal and visual memory [53, 54] in LLD, but not in HC [53]. In their LOD vs. HC study, Lim et al. (2012) concluded that lower cortical thickness of the left superior temporal, precuneus, entorhinal cortex, and isthmus cingulate, correlated with lower verbal memory scores [44]. Additional regions included the fusiform, insula, precentral, and supramarginal [44]. However, another VBM study in patients with remitted LOD reported a contradictory association between volume of the cingulate gyrus and episodic memory, whereby larger volumes were correlated with worse memory functioning [48].

Both cortical and subcortical abnormalities in frontal-executive regions were specifically associated with poor verbal memory. Volume loss in subcortical regions, including the putamen, thalamus, and anterior caudate, was significantly associated with worse verbal recall in LLD [44, 55] but not HC [44, 55]. Similarly, volume loss in the rostral middle frontal, medial frontal, and caudal anterior cingulate as well as lower cortical thickness of inferior temporal and inferior parietal cortex were also associated with deficits in verbal memory in LLD [44, 53, 56]. In addition to LLD or HC groups, one single study included aMCI and LLD+aMCI groups [23]. There were no associations between GM volumes and episodic memory in any of the groups. However, the sample size was small with <20 participants in each case group.

Finally, Shin et al. (2018) used a structural network correlation analysis in 50 participants with untreated LLD. They reported that a sub-network with cores belonging to the left subcentral cortex, right precuneus, and the posterior ramus of the right lateral sulcus was correlated with verbal fluency scores (but not episodic memory) [45]. Single univariate associations were also identified between lower cortical thickness of the fusiform or lateral occipital gyri and worse episodic memory function, which agreed with other studies [45].

#### T1-weighted MRI comparisons of EOD vs. LOD

While nine T1 studies included both EOD and LOD groups [33, 35–37, 42, 49, 51, 55, 57], only 4 of them compared them in terms of brain-cognition relationships (with all 4 studies including participants with current depression) [35–37, 49]. The results of three studies support associations between GM thickness or volume and poor cognition in LOD, but not EOD or HC [35, 36, 49]. In the fourth study, conversely, whole-brain and bilateral frontal lobe volumes were associated with global cognition in participants with EOD or HC but not in those with LOD [37].

### DWI studies

Eighteen of the 51 studies used DWI to assess the relationships between WM tract integrity measures (fractional anisotropy (FA), mean diffusivity (MD), radial diffusivity (RD), or axial diffusivity (AD)) and cognition in LLD. Six studies used voxel-wise TBSS analysis, 11 studies used an ROI approach (of which 3 used tractography) and 2 studies used network-based statistics (NBS) (Table 3). Two studies included additional groups: an aMCI and LLD+aMCI groups [21] or an additional LLD with memory deficits group [58]. Eleven of the 18 DWI studies included participants with acute depression, 6 included participants with remitted depression, and one study included a mixed sample (i.e., remitted + current).

#### TBSS studies (voxel-wise analysis)

##### Executive functioning and processing speed

Six studies used a voxel-wise analysis to explore the association of whole-brain WM tract integrity with executive function and processing speed. Of those, 4 studies reported positive associations in the LLD group [7, 59–61], 1 study in the HC group only [62], and 1 study reported no associations in any group [63]. The results of studies with similar analytic approaches that identified a significant relationship with WM integrity included associations with some specific cognitive tests but not with others. For example, in one study, decreased FA in a cluster including the superior longitudinal fasciculus (SLF), SLF-temporal, and right corticospinal tract in the LLD group correlated with lower scores on the initiation/perseveration subscale of the dementia rating scale (DRS), but not with the Stroop Color Word Interference (CWI) task [59]. In a comparable study, lower FA in fronto-striatal-limbic tracts in the LLD group was correlated with the Stroop CWI Task [60]. One other study in remitted LLD showed that lower FA of the anterior thalamic radiation and the uncinate fasciculus was correlated with executive dysfunction, and lower FA of the genu of the corpus callosum was correlated with impaired processing speed [7].

##### Learning and episodic, verbal, or visuospatial memory

Two of the 4 DWI studies that assessed the association between WM tract integrity and performance on learning and memory tasks in LLD identified significant associations [7, 63], while two did not [59, 61]. Poor tract integrity (i.e., lower FA) in corticolimbic circuitry [63] and the genu/body of the corpus callosum and fornix [7] were associated with poor episodic memory. Alves et al. (2012) found an association between lower FA of the right posterior cingulate cluster and poor verbal fluency and episodic memory in their whole sample (LLD + HC). No significant associations were reported in the HC group [63].

#### Region of interest analysis

##### Executive function and processing speed

Of the 11 studies using an ROI approach, 10 assessed the relationships between WM tract integrity and executive function or processing speed. Five focused on the associations between corticolimbic tract integrity (i.e., the cingulate bundle and uncinate fasciculus) and executive function or processing speed in LLD [62, 64–67], with 4 reporting positive associations [62, 65–67]. One study selected cingulum bundle fiber tracts connecting the PCC/precuneus to the dorsal ACC, for which lower FA was associated with executive dysfunction across the whole sample (LOD + HC) [66]. This finding implicating the posterior cingulate bundles was replicated in another study of remitted LOD [67]. Higher integrity of the corpus callosum (lower MD) was associated with faster processing speed [68], but this finding was not replicated in another study with a similar regional analysis [65], potentially due to the remission status in their mixed sample of remitting and non-remitting participants [65]. Regions of interest found in other studies included the anterior and posterior commissure and the anterior thalamic radiation, in which lower tract integrity (low FA) was associated with executive dysfunction [69, 70]. Finally, no brain-cognition associations with white matter integrity were found in remitted LOD [71]. Despite the heterogeneity in findings, there is an overall trend of an association between lower white matter tract integrity and worse cognition.

##### Learning and episodic, verbal, or visuospatial memory

Eight DWI studies examined the relationships between integrity of selected WM tracts with learning and memory in LLD [21, 64–68, 70, 71]. Six of these studies reported no significant brain-cognition associations [64, 66–68, 70, 71]. Only two studies reported significant associations [21, 65]. In the first, lower FA of the cingulate and uncinate fasciculus was associated with poor episodic memory and language; however, this correlation was weaker than that with executive function or processing speed [65]. The second study reported associations between higher diffusivity (i.e., MD and RD), but not lower anisotropy, values of the cingulum-hippocampus tract and poor episodic memory across patients with LLD, aMCI, LLD+aMCI, and HC [21]. However, they did not find any significant associations within each case group [21].

#### Additional approaches: network-based statistics and machine learning

Two DWI studies used probabilistic tractography and network-based analysis (NBS) to explore the relationship between WM connectivity and cognition in remitted LLD [72], LOD, or LOD + memory deficits [58]. In the first study, less connectivity in a subnetwork comprising the left lingual gyrus, middle occipital gyrus, and fusiform gyrus was associated with slowed processing speed in the remitted LLD group, but not in HC [72]. In the other study, altered connectivity in a global subnetwork spanning frontal, temporal, parietal, and subcortical regions in LOD [58] was associated with executive function, processed speed, and memory, with the highest correlation with processing speed [58].

A third DWI study used relevance vector regression (RVR), a multivariate machine learning approach, to predict the contribution of multimodal MRI features (i.e., T1 and DWI) to slowed processing speed in remitted LOD patients [73]. Their model identified 26 GM volumes and 8 WM features (3 FA and 5 MD), whereby lower GM volumes and lower tract integrity predicted worse processing speed [73].

## Discussion

We conducted a systematic review of 51 MRI studies (33 T1 studies, 17 DWI studies, and 1 study analyzing both T1 and DWI) of brain-cognition relationships in patients with LLD. Three main findings emerge from these studies, including consistent gray matter regions and white matter circuitry implicated in cognitive impairment in LLD. First, overall, these studies support the role of altered corticolimbic circuitry (particularly in the hippocampus, precuneus, entorhinal cortex, and cingulate cortex) in deficits of learning and memory. DWI studies more consistently implicate the cingulate bundle and other posterior cingulate clusters including the corpus callosum sub-regions in executive dysfunction. Second, measures of WM integrity were more strongly correlated with executive dysfunction than with memory impairments. Third, more consistent brain-cognition relationships emerged for LOD than EOD.

Confidence in these results is tempered by methodological limitations in the literature and in our review. The relevant studies we identified were heterogeneous in terms of both participant profiles and imaging methods. For example, LLD participants varied in depression status, age of onset, or treatment status. Also, most studies excluded patients with LLD and comorbid MCI or dementia based on their MMSE scores. However, the MMSE lacks sensitivity to cognitive deficits in patients with LLD [74]. Thus, cognitive impairment in patients with LLD in these studies may contribute to the heterogeneity of results. Similarly, our discussion of relationships between brain structure and global cognition is limited by the prevalent use of the MMSE as a measure of global cognition, which presents with major limitations (e.g., poor test-retest reliability) as an accurate measure of interindividual variability in cognition [75]. Another limitation of the surveyed literature is the relatively small sample sizes, ranging from 12 to 99 participants (with the exception of one study [31]). These findings should be replicated in future studies with larger sample sizes featuring hundreds of participants to identify replicable brain-behavior associations [76, 77]. Moreover, we identified only one T1 study [23] and one DWI study that compared LLD to MCI or LLD + MCI; more studies should compare participants with LLD and varying cognitive profiles (including LLD + MCI). Findings from those two studies should also be replicated in future studies with larger sample sizes, given the small sizes of each group (ranging from 12 to 33). Moreover, while seven studies included a longitudinal component, except for two short clinical trials, none of the longitudinal studies systematized antidepressant treatment, making it impossible to assess the effect of time vs. treatment. Lastly, our review focused on structural MRI, and did not address functional neuroimaging modalities (e.g., functional MRI or positron emission tomography imaging); therefore, we cannot comment on the relationships between functional brain activity and cognitive function. Notwithstanding these limitations, some of the broad themes emerging from the reviewed studies deserve further discussion.

### The cingulate region is a potential treatment target for LLD and LLD + MCI

One theme consistent across both T1 and DWI studies is the regional involvement of the cingulate (anterior and posterior sub-regions) region in impaired cognition in LLD in general [46, 53, 60, 63] and LOD in particular [66, 67]. The ACC region belongs to the salience network and has a unique role in emotional regulation and cognitive function due to connections to limbic (e.g., amygdala, hippocampus, striatum) and prefrontal cortex (e.g., DLPFC) areas [78]. It has been suggested that emotional disturbances, particularly apathy, and cognitive deficits, particularly executive dysfunction, in LLD could share neurobiological mechanisms including poor white matter integrity of the ACC [17]. The PCC is a corticolimbic structure and a core region of the default mode network (DMN) with connections to key regions implicated in memory such as the hippocampus, parahippocampus, and entorhinal cortex [79]. This finding is congruent with results of several functional connectivity studies, which have consistently reported altered functional connectivity within the DMN in patients with LLD [80, 81], MCI + LLD [80], or AD with depressive symptoms [82]. Moreover, this agrees with previous findings from our group indicating that posterior DMN regions (PCC and precuneus) consistently showed structural and functional alterations in LLD, MCI, and LLD + MCI [22]. In a recent systematic review of 14 deep brain stimulation (DBS) clinical trials in patients with treatment-resistant depression, targeting the subcallosal cingulate cortex appeared to have a promising antidepressant effect [83, 84]. Taken together, findings from this review focused on depression and findings from studies focusing on MCI [85] suggest that the cingulate region is a region with shared vulnerability to both depression and cognitive impairment. It could be an intervention target for brain stimulation to mitigate the risk of AD associated with LLD or MCI.

### White matter integrity is primarily associated with executive function

WM tract integrity was more strongly associated with executive function or processing speed, than learning or memory. Eight of the 10 reviewed DWI ROI studies found an association between WM tract integrity and executive function or processing speed [62, 65–70, 73]. By contrast, of the DWI studies, 6/8 ROI [64, 66–68, 70, 71] and 2/4 TBSS studies [59, 61] did not find an association between WM integrity and memory or learning. This finding holds true across different studies and within studies that tested different cognitive domains alongside each other [59, 65, 70]. This finding in LLD is congruent with studies in healthy older adults [86–88]. This suggests that degradation of WM integrity associated with normal aging or LLD drives the observed higher-order cognitive decline in executive function and processing speed.

### LOD is a potential marker for cognitive decline

Lastly, stronger brain-cognition relationships were observed in LOD than EOD or non-depressed controls. This result extends findings from our meta-analysis of structural MRI studies showing more abnormalities in the gray matter of fronto-parietal, dorsal attention, and visual networks in LOD than in those of EOD or mixed-onset LLD [89]. Similarly, in a longitudinal study of hippocampal volumes, participants with LOD experienced hippocampal atrophy at a faster rate than those with EOD [14]. Other studies have also reported a strong association between white matter hyperintensities (WMH) in fronto-striatal circuits and cognitive decline in LOD [15]. These findings support the hypothesis that LOD is a form of “vascular depression” and a prodrome for dementia [17, 90, 91]. The differences in brain-cognition relationship between LOD and EOD in the studies we reviewed are consistent with this hypothesis.

## Conclusions

Our review of 51 studies of brain-cognition relationships using T1 and DWI measures provides evidence of brain circuitry that could differentially underlie cognitive impairment in LLD. Our analysis of longitudinal T1 studies highlights the role of the hippocampus in global cognition and other domains of memory in LLD, while our cross-sectional analysis highlights the role of additional corticolimbic regions including the cingulate cortex in learning and memory. Findings from the DWI studies we reviewed implicate white matter integrity of the cingulate bundle sub-regions with executive dysfunction. Our results highlight gray matter regions and white matter circuitry with a shared vulnerability to both LLD and cognitive impairment and summarize structural brain circuitry vulnerable to cognitive impairment in LLD. Our results may inform the design of preventive interventions for patients at risk of developing AD.

### Future directions

While a reasonable number of studies have explored the relationship between GM or WM and cognitive impairment in LLD, many questions remain unaddressed in this field. Future studies should include and contrast participants with LLD and with varying degrees of cognitive impairment to determine whether brain-cognition relationships differ between LLD and LLD + MCI. In our review, only 4 studies compared brain-cognition relationships in EOD versus LOD; future studies should stratify their analysis according to depression age of onset. Also, most of the GM studies and all the WM studies we reviewed were cross-sectional; future longitudinal studies using multi-modal imaging that control for antidepressant treatment are needed to better understand causal relationships between alterations in brain circuitry and cognitive impairment. These future studies will need larger sample sizes to identify reproducible brain-behavior associations [92]. By uncovering altered brain circuitry and cognition in specific subgroups of patients with LLD and varying degrees of cognitive impairment, these future studies will inform the design of strategies to prevent AD.

### Supplementary information

Supplementary Materials File

## Data Availability

The raw data supporting the conclusions of this article will be made available by the authors, without undue reservation, to any qualified researcher.
