# Supplementary material for: Brain-cognition relationships in late-life depression: a systematic review of structural magnetic resonance imaging studies
Source: Transl Psychiatry. 2023 Aug 19;13:284. doi: 10.1038/s41398-023-02584-2 (PMC10439902; doi:10.1038/s41398-023-02584-2)
Supplement: Supplementary file 1 — Supplementary Materials File [file 41398_2023_2584_MOESM1_ESM.docx]

**Supplementary Material**

**Supplementary Table 1. Search Strategy Adapted for MEDLINE**

| **Search Block 1: Depression (late-life)** | **Search Block 2: MRI** | **Search Block 3: Cognition** |
| --- | --- | --- |
| 1. exp Depressive Disorder/ | 11. neuroimaging/ or diffusion tensor imaging/ | 22. Cognition/ or cognit*.mp. |
| 2. exp Depression/ | 12. magnetic resonance imaging/ or diffusion magnetic resonance imaging/ | 23. (neuropsych* or neuro-psych*).mp |
| 3. (depress* adj3 (disorder* or diagnose*)).mp. | 13. (neuroimag* or neuro-imag* or diffusion tensor imaging or magnetic resonance imaging or diffusion magnetic resonance imaging).mp. | 24. (neurocog* or neuro-cog*).mp. |
| 4. 1 or 2 or 3 | 14. ((T1-weighted or T1 weighted) adj2 (imag* or scan or MRI)).mp. | 25. exp Executive Function/ or executive function*.mp. |
| 5. exp Geriatrics/ | 15. white matter.mp. or exp White Matter/ | 26. exp Memory/ or memory.mp. |
| 6. (geriatric* or older adult* or elderly or aging or late-life or late life or "late in life" or late-onset).mp. | 16. fractional anisotropy.mp. | 27. 22 or 23 or 24 or 25 or 2 |
| 7. 5 or 6 | 17. mean diffusivity.mp. | 28. 10 and 21 and 27 |
| 8. 4 and 7 | 18 grey matter.mp. or exp Gray Matter/ |  |
| 9. ((late-life or late-onset or "late in life" or geriatric) adj3 depress*).mp. | 19. cortical thickness.mp. |  |
| 10. 8 or 9 | 20. ((subcortical or sub-cortical) adj2 volume*).mp. |  |
|  | 21. 11 or 12 or 13 or 14 or 15 or 16 or 17 or 18 or 19 or 20 |  |

**Supplementary Table 2. Search Strategy Adapted for EMBASE**

| 1 | depression/ or exp late life depression/ or exp major depression/ |
| --- | --- |
| 2 | (depress* adj3 (disorder* or diagnos*)).mp. |
| 3 | 1 or 2 |
| 4 | geriatric.mp. or exp geriatrics/ |
| 5 | (geriatric* or older adult* or elderly or aging or late-life or late life or "late in life" or late-onset).mp. |
| 6 | 4 or 5 |
| 7 | 3 and 6 |
| 8 | ((late-life or late-onset or "late in life" or geriatric) adj3 depress*).mp. |
| 9 | 7 or 8 |
| 10 | exp neuroimaging/ or neuroimaging.mp. |
| 11 | nuclear magnetic resonance imaging/ or diffusion tensor imaging/ or diffusion weighted imaging/ |
| 12 | (neuroimag* or neuro-imag* or diffusion tensor imaging or diffusion weighted imaging or magnetic resonance imaging).mp. |
| 13 | ((T1-weighted or T1 weighted) adj2 (imag* or scan or MRI)).mp. |
| 14 | white matter.mp. or exp white1- matter/ |
| 15 | fractional anisotropy.mp. or exp fractional anisotropy/ |
| 16 | mean diffusivity.mp. or exp mean diffusivity/ |
| 17 | grey matter.mp. or exp gray matter/ |
| 18 | exp "cortical thickness (brain)"/ or cortical thickness.mp. |
| 19 | ((subcortical or sub-cortical) adj2 volume*).mp. |
| 20 | 10 or 11 or 12 or 13 or 14 or 15 or 16 or 17 or 18 or 19 |
| 21 | cognition.mp. or exp cognition/ |
| 22 | (neuropsych* or neuro-psych*).mp. |
| 23 | (neurocog* or neuro-cog*).mp. |
| 24 | executive function.mp. or exp executive function/ |
| 25 | exp memory/ or memory.mp. |
| 26 | 21 or 22 or 23 or 24 or 25 |
| 27 | 9 and 20 and 26 |

**Supplementary Table 3. Search Strategy Adapted for PsycINFO**

| 1 | major depression/ or late life depression/ |
| --- | --- |
| 2 | (depress* adj3 (disorder* or diagnos*)).mp. |
| 3 | 1 or 2 |
| 4 | geriatric.mp. or exp Geriatrics/ |
| 5 | (geriatric* or older adult* or elderly or aging or late-life or late life or "late in life" or late-onset).mp. |
| 6 | 4 or 5 |
| 7 | 3 and 6 |
| 8 | ((late-life or late-onset or "late in life" or geriatric) adj3 depress*).mp. |
| 9 | 7 or 8 |
| 10 | magnetic resonance imaging/ or neuroimaging/ or diffusion tensor imaging/ |
| 11 | (magnetic resonance imaging or neuroimag* or neuro-imag* or diffusion tensor imaging).mp. |
| 12 | ((T1-weighted or T1 weighted) adj2 (imag* or scan or MRI)).mp. |
| 13 | ((diffusion-weighted or diffusion weighted) adj2 (imag* or scan or MRI)).mp. |
| 14 | white matter.mp. or exp White Matter/ |
| 15 | fractional anisotropy.mp. |
| 16 | mean diffusivity.mp. |
| 17 | grey matter.mp. or exp Gray Matter/ |
| 18 | cortical thickness.mp. |
| 19 | ((subcortical or sub-cortical) adj2 volume*).mp. |
| 20 | 10 or 11 or 12 or 13 or 14 or 15 or 16 or 17 or 18 or 19 |
| 21 | exp Cognition/ or cognition.mp. or cognit*.mp. |
| 22 | (neuropsych* or neuro-psych*).mp. |
| 23 | (neurocog* or neuro-cog*).mp. |
| 24 | executive function.mp. or exp Executive Function/ |
| 25 | memory.mp. or exp Memory/ |
| 26 | 21 or 22 or 23 or 24 or 25 |
| 27 | 9 and 20 and 26 |

**Supplementary Table 4. Search Strategy Adapted for Web of Science**

| **1** | (((“late-life” or “late-onset” or “late in life” or “geriatric” ) NEAR/3 depress*))  *Indexes = SCI-EXPANDED, SSCI, A&HCI, CPCI-S, CPCI-SSH, BKCI-S, BKCI-SSH, ESCI Timespan = All years* |
| --- | --- |
| **2** | (neuroimage* OR neuro-imag*)  *Indexes = SCI-EXPANDED, SSCI, A&HCI, CPCI-S, CPCI-SSH, BKCI-S, BKCI-SSH, ESCI Timespan = All years* |
| **3** | (“magnetic resonance imaging” OR “MRI)  *Indexes = SCI-EXPANDED, SSCI, A&HCI, CPCI-S, CPCI-SSH, BKCI-S, BKCI-SSH, ESCI Timespan = All years* |
| **4** | (diffusion NEAR/3 (imag* OR scan OR MRI))  *Indexes = SCI-EXPANDED, SSCI, A&HCI, CPCI-S, CPCI-SSH, BKCI-S, BKCI-SSH, ESCI Timespan = All years* |
| **5** | (T1 NEAR/3 (imag* OR scan OR MRI))  *Indexes = SCI-EXPANDED, SSCI, A&HCI, CPCI-S, CPCI-SSH, BKCI-S, BKCI-SSH, ESCI Timespan = All years* |
| **6** | ((white OR grey OR gray) NEAR/3 matter)  *Indexes = SCI-EXPANDED, SSCI, A&HCI, CPCI-S, CPCI-SSH, BKCI-S, BKCI-SSH, ESCI Timespan = All years* |
| **7** | ((cortical OR subcortical) NEAR/3 (thickness OR volume))  *Indexes = SCI-EXPANDED, SSCI, A&HCI, CPCI-S, CPCI-SSH, BKCI-S, BKCI-SSH, ESCI Timespan = All years* |
| **8** | (“fractional anisotropy” OR “mean diffusivity”)  *Indexes = SCI-EXPANDED, SSCI, A&HCI, CPCI-S, CPCI-SSH, BKCI-S, BKCI-SSH, ESCI Timespan = All years* |
| **9** | 8 or 7 or 6 or 5 or 4 or 3 or 2  *Indexes = SCI-EXPANDED, SSCI, A&HCI, CPCI-S, CPCI-SSH, BKCI-S, BKCI-SSH, ESCI Timespan = All years* |
| **10** | (cognit* OR neuropsych* OR neurocog*)  *Indexes = SCI-EXPANDED, SSCI, A&HCI, CPCI-S, CPCI-SSH, BKCI-S, BKCI-SSH, ESCI Timespan = All years* |
| **11** | 10 and 9 and 1  *Indexes = SCI-EXPANDED, SSCI, A&HCI, CPCI-S, CPCI-SSH, BKCI-S, BKCI-SSH, ESCI Timespan = All years* |

**Supplementary Table 5. Newcastle-Ottawa Scale (NOS) Scores of Studies with Both Late-Life Depression (LLD) and Healthy Controls (HC) Groups**

| **Reference** | **LLD (n)** | **HC (n)** | **Image Modality** | **NOS Score** |
| --- | --- | --- | --- | --- |
| Ballmaier et al. (2008) | 28 | 15 | T1 | 7 |
| Lai et al. (2000) | 20 | 20 | T1 | 5 |
| Lim et al. (2012) | 48 | 47 | T1 | 8 |
| Chang et al. (2011) | 88 | 35 | T1 | 6 |
| Steffens et al. (2003) | 30 | 40 | T1 | 7 |
| Egger et al. (2008) | 14 | 20 | T1 | 7 |
| Almeida et al. (2003) | 51 | 37 | T1 | 5 |
| Elderkin-Thompson et al. (2009) | 26 | 23 | T1 | 6 |
| Yuan et al. (2008) | 19 | 16 | T1 | 6 |
| Lamar et al. (2012) | 18 | 33 | T1 | 5 |
| Avila et al. (2011) | 48 | 31 | T1 | 6 |
| Colloby et al. (2011) | 38 | 30 | T1 | 7 |
| Lebedeva et al. (2015) | 49 | 49 | T1 | 6 |
| Steffens et al. (2000) | 66 | 18 | T1 | 5 |
| Shin et al. (2018) | 50 | 48 | T1 | 8 |
| Sheline et al. (2008) | 83 | 32 | T1 | 7 |
| Jayaweera et al. (2016) | 84 | 27 | T1 | 5 |
| Lloyd et al. (2004) | 51 | 39 | T1 | 6 |
| Choi et al. (2017) | 50 | 50 | T1 | 7 |
| Ashtari et al. (1999) | 40 | 46 | T1 | 5 |
| Bell-McGinty et al. (2002) | 30 | 47 | T1 | 5 |
| Pantel et al. (1997) | 19 | 13 | T1 | 5 |
| Greenwald et al. (1997) | 30 | 36 | T1 | 4 |
| Sexton et al. (2012) | 36 | 25 | T1 and DWI | 6 |
| Sawyer et al. (2012) | 238 | 146 | T1 | 6 |
| Steffens et al. (2011) | 90 | 72 | T1 | 6 |
| Marano et al. (2015) | 17 | 17 | T1 | 6 |
| Hou et al. (2011) | 14 | 19 | T1 | 3 |
| Kohler et al. (2010) | 35 | 29 | T1 | 6 |
| Yuan et al. (2007) | 16 | 14 | DWI | 5 |
| Lamar et al. (2013) | 26 | 34 | DWI | 7 |
| Alves et al. (2012) | 17 | 18 | DWI | 6 |
| Yuan et al. (2010) | 37 | 33 | DWI | 6 |
| Charlton et al. (2014) | 23 | 23 | DWI | 7 |
| Shimony et al. (2009) | 73 | 23 | DWI | 7 |
| Mettenburg et al. (2012) | 51 | 16 | DWI | 5 |
| Yin et al. (2016) | 32 | 39 | DWI | 7 |
| Wang et al. (2020) | 37 | 30 | DWI | 5 |
| Wang et al. (2021) | 40 | 36 | DWI | 6 |
| Li et al. (2017) | 24 | 24 | DWI | 5 |
| Zhou et al. (2022) | 74 | 68 | DWI | 7 |

DWI: diffusion-weighted imaging

**Supplementary Table 6. Newcastle-Ottawa Scale (NOS) Scores of Studies Only with Late-Life Depression (LLD) Group**

| **Reference** | **LLD (n)** | **Image Modality** | **NOS Score** |
| --- | --- | --- | --- |
| Yuan et al. (2010) | 37 | T1 | 4 |
| Droppa et al. (2017) | 26 | T1 | 4 |
| Sachs-Erisson et al. (2011) | 61 | T1 | 3 |
| Dahabra et al. (1998) | 17 | T1 | 6 |
| He et al. (2021) | 71 | DWI | 4 |
| Murphy et al. (2007) | 51 | DWI | 5 |
| Alexopoulos et al. (2002) | 13 | DWI | 3 |

DWI: diffusion-weighted imaging

**Supplementary Table 7. Newcastle-Ottawa Scale (NOS) Scores of Studies with Late-Life Depression (LLD), and Other Groups**

| **Reference** | **LLD (n)** | **HC (n)** | **MCI (n)** | **LLD+MCI (n)** | **Image Modality** | **NOS Score** |
| --- | --- | --- | --- | --- | --- | --- |
| Xie et al. (2012) | 18 | 25 | 17 *aMCI | 12 | T1 | 7 |
| Li et al. (2013) | 20 | 33 | 18 *aMCI | 13 | DWI | 7 |
| Mai et al. (2017) | 24 | 30 | - | 15 *LLD+MD | DWI | 6 |

aMCI: amnestic Mild-Cognitive Impairment; DWI: diffusion-weighted imaging; HC: healthy controls; MD: memory deficit (no formal MCI diagnosis)
